# Persistently elevated intracranial pressure in cryptococcal meningitis– 76 therapeutic lumbar punctures

**DOI:** 10.1016/j.mmcr.2023.04.001

**Published:** 2023-04-28

**Authors:** Samuel Jjunju, Edwin Nuwagira, David B. Meya, Conrad Muzoora

**Affiliations:** a Department of Internal Medicine, Mbarara University of Science and Technology, Mbarara, Uganda; b Research Department, Infectious Disease Institute, Makerere University, Kampala, Uganda

**Keywords:** Cryptococcal meningitis, Increased intracranial pressure, Lumbar puncture, Human Immunodeficiency Virus

## Abstract

Cryptococcal meningitis still remains the most common form of adult meningitis in sub-Saharan Africa, due to the burden of HIV/AIDS. Increased intracranial pressure (ICP) is a major complication of cryptococcosis and requires aggressive management with therapeutic lumbar punctures (LPs). In this report, we describe a patient with persistently elevated ICP who underwent 76 LPs over 46 days with good outcome. While unusual, this highlights the importance of serial therapeutic LPs.

2012 Elsevier Ltd. All rights reserved.

## 1 Introduction

HIV-associated cryptococcal meningitis is the most common form of meningitis diagnosed among adults with HIV in sub-Saharan Africa [1], despite expanded access to antiretroviral therapy (ART). Cryptococcus remains a leading cause of death among people with HIV/AIDS in resource-limited settings and is responsible for 15–20% of all AIDS-related deaths globally [1].

Raised intracranial pressure (ICP) is one of the common complications of cryptococcosis, defined as cerebrospinal fluid (CSF) opening pressure >20cm H2O [2,3]. Elevated, uncontrolled ICP is associated with poor prognosis in cryptococcal meningitis [2,4,5]. Raised ICP may occur at the time of diagnosis or develop during the course of treatment and can also occur as part of an immune reconstitution inflammatory syndrome (IRIS). Raised ICP may result in worsening headaches, vomiting, changes in mental status, loss of vision and hearing, seizures and death [4,6].

Aggressive management of raised ICP is recommended in WHO cryptococcal treatment guidelines [7]. This includes daily therapeutic lumbar punctures (LPs) until pressures normalize and symptoms resolve [4,7,8]. In the Cryptococcal Optimal ART Timing (COAT) trial in Uganda and South Africa [9], individuals that received at least one therapeutic lumbar puncture in the first week had a 69% greater chance of survival, regardless of initial CSF opening pressure, when compared with individuals who did not receive therapeutic LPs [4].

There are different methods of reducing ICP which include therapeutic LPs, lumbar drains, ventriculostomy, or ventriculo-peritoneal shunts [7,8,10]. Lumbar drains and VP shunts are more commonly used in high-income countries [8]. In resource-limited settings, repeated therapeutic LPs are most commonly used for the management of raised ICP [3]. There are potential complications associated with LPs and repeated LPs including brain herniation, and bacterial arachnoiditis or meningitis, but these are probably very rare [11], and, in the setting of HIV-associated cryptococcal meningitis, far outweighed by the mortality benefit of CSF pressure control.

We present our experience with a participant of the AMBIsome Therapy InductioN OptimisatioN trial (AMBITION ISRCTN: 72509687) [12] who was diagnosed with cryptococcal meningitis complicated by persistently raised ICP and who underwent 76 therapeutic LPs over a period of 46 days. Written informed consent was obtained for both the trial and this case report.

## 2 Case presentation

A 23-year old female living with HIV infection was admitted to Mbarara Regional Referral Hospital (MRRH) in Mbarara, Uganda where she presented with a two-week history of severe headache associated with a 10 day history of generalized tonic-clonic seizures, blurring of vision, hearing loss, vomiting and low-grade fevers. She had a one-month history of productive cough with associated night sweats and loss of appetite but no loss of weight. She was not confused, and review of other systems was unremarkable. At time of admission, her HIV viral load and CD4 count were unknown. She had previously been on an antiretroviral therapy (ART) regimen of tenofovir, lamivudine, and efavirenz for three years, which had been changed two months prior admission, to tenofovir, lamivudine, and dolutegraviras part of the revised Ugandan HIV treatment guidelines [13] and reported good drug adherence.

On general examination, she was sick-looking (lethargic and lying supine in bed) with wounds on the tongue but no pallor, jaundice, edema, cyanosis, dehydration, lymphadenopathy or skin lesions. Her vitals were: blood pressure 113/78 mmHg, pulse rate 98 beats per minute, temperature 36.0 °C, and respiratory rate 20 breaths per minute. She was fully conscious (Glasgow coma scale score 15) with positive Kernig's and Brudzinski's signs, had a right eye cranial nerve VI palsy, and bilateral dilated pupils which were sluggishly reactive to light. Visual acuity for the right eye was 0.700 and for the left eye 0.600 using the Sonksen logMAR Test- Near chart. There were no other neurological findings and the rest of her systemic examination was normal.

A bedside finger prick blood cryptococcal antigen (CrAg) lateral flow assay (IMMY, Norman, Oklahoma, USA) was positive. A baseline brain computed tomography (CT) scan was not carried out, since having cranial nerve VI palsy was not one of the contraindications for executing an LP in the AMBITION-cm study [14,15]. A diagnostic LP was performed and revealed clear CSF with an opening pressure of 7 cmH_2_O, and 7 mL of CSF was removed for analysis, culture, and storage. A bedside CSF CrAg was positive and CSF glucose was 4.6mmol/L.

Baseline CSF analysis results were as follows: total white blood cell count 16 cells/mm^3^ (78% lymphocytes, 22% neutrophils), a gram stain showed yeast cells, India Ink was positive, protein 10mg/dL and a quantitative Cryptococcal culture of 2,400,000 colony forming units (CFU) per mL of CSF. Baseline chemistry and hematology were within normal limits. A CD4 count was 6 cells/uL, and HIV viral load was 3302 copies/mL. Sputum Xpert MTB/Rif (Cepheid, Sunnyvale, CA) and urine lipoarabinomannan (LAM) (Alere, Waltham, MA) for *Mycobacterium tuberculosis* were negative. A chest radiograph was unremarkable.

The patient received induction therapy with intravenous amphotericin B deoxycholate 1mg/kg/day for 7 days plus oral flucytosine 100mg/kg/day in four divided doses for 7 days followed by 7 days of fluconazole 1200 mg/day. The patient also received potassium and magnesium supplementation, sodium valproate to control the seizures, and continued ART.

**Fig. 1:**
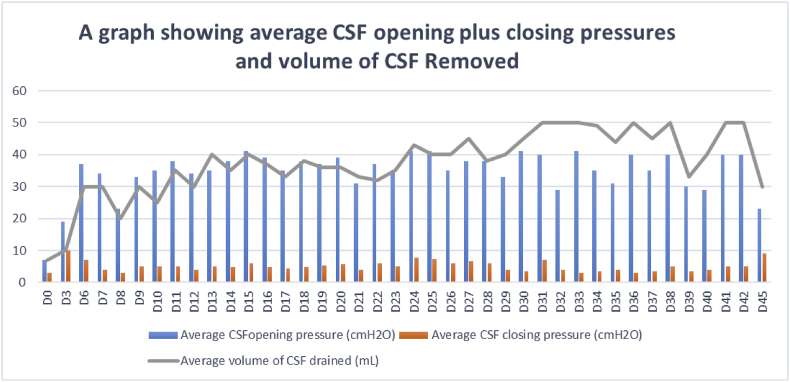
A graph representing trend in average daily CSF opening plus closing pressures and volume of CSF drained.

On day six of hospitalization, she developed symptoms of raised ICP: vomiting, severe headache and convulsions. All these symptoms had ceased since admission. A therapeutic LP identified a raised opening pressure of 37 cmH_2_O with 30mL of CSF being drained [15]. Serial therapeutic LPs were performed however the patient had recurrent headaches, and CSF opening pressures were persistently elevated. On every therapeutic LP, closing pressures were rechecked after removal of every 10mls of CSF and on some occasions pressures would be above 20cmH2O after removal of 40mls of CSF and patient would still complain of headache and this prompted the study clinician to remove more than 40mls of CSF deviating from the AMBITION-cm study guidelines on management of raised ICP [15] however after that the patient would report relief and also the closing pressures would come to less than 20cmH2O. Fig. 1 shows average daily CSF opening plus closing pressures and volume of CSF removed over a period of 43 days.

A total of 76 LPs were performed during the patient's entire admission, the frequency which of was guided by the signs and symptoms (headache, vomiting and convulsions) of raised ICP and the opening pressure of the previous LP [15]. The participant regularly called the research team, often in the night, to request a LP. Follow up CSF cultures were sterile at both day 7 and 14. A contrasted Brain CT scan was done on day 14 which showed dilated ventricles, however no cryptococcomas or other space occupying lesions were noted.

Patient outcomes were good. Their visual acuity at week 10 was 0.200 in the left eye and 0.300 in the right eye, which was a marked improvement from baseline. Elevated ICP normalized with resolution of signs and symptoms of raised ICP, and the patient was discharged on day 46 of hospitalization. They completed consolidation phase fluconazole 800mg/day through 10 weeks, and then maintenance dose fluconazole 200mg/day. She was actively followed through 16 weeks and successfully linked to a primary HIV clinic for follow up and ongoing care.

## 3 Discussion

Elevated ICP is one of the most common complications of cryptococcal meningitis reported in >50% of patients [2,13]. In cryptococcosis, the pathophysiology is believed to be caused by obstruction of the reabsorption of CSF at the arachnoid villi by *Cryptococcus* leading to a communicating hydrocephalus [16]. This case shows the importance of detecting raised ICP during the course of the illness and the safety of aggressive management with serial therapeutic LPs. Elevated ICP increases the morbidity and mortality of HIV-associated cryptococcal meningitis, especially if not properly managed [4,14]. Normal baseline opening pressure does not rule out the risk of developing raised ICP as CSF pressure can build up over time during the course of treatment [4,17]. In this case, the patient had normal baseline opening pressure; however, by day 7, pressure had built up to a level sufficient to cause symptoms (headache, vomiting, and convulsions) and then persisted for much longer than usual.

Based on recurrence of symptoms, management at times required multiple LPs on a single day for pressure control. This patient had a high baseline CSF fungal burden, and this delayed onset of raised ICP is likely due to the antifungal therapy precipitating the death of a large amount of *Cryptococcus* and capsule, which later obstructed the arachnoid villi [18]. The diameter of the *Cryptococcus* capsule is positively associated with increased intracranial pressure [18], and whether the yeast are dead or alive, obstruction of arachnoid villi can still occur. In addition, it is possible, despite reported adherence to ART, prior to admission, some degree of IRIS, on top of the very high organism load at baseline, may have contributed to the raised ICP in this case.

There are three common methods used to control persistent, severely raised ICP in cryptococcal meningitis: therapeutic LPs, lumbar drains, and ventriculoperitoneal shunts. However, in resource-limited settings serial LPs are the most common and often the only method of lowering raised ICP. Previous studies have explored pharmacological treatments for raised ICP including acetazolamide had no therapeutic benefit and was associated with more frequent serious adverse events [19].

Active management of raised ICP is important as it has been shown to be associated with improved patient outcomes and reduced mortality [4]. Outdated Infectious Disease Society of America (IDSA) guidelines [8], recommend therapeutic LPs if the previous opening pressure is > 25 cm H2O and there are patient symptoms of increased ICP. Others have advocated for scheduled LPs regardless of initial baseline pressure, due to the survival benefit of repeat therapeutic LP in the first week [4,12]. In this case, the number of LPs the patient received in a day ranged from 1 to 4. The patient consistently reported an immediate symptomatic relief, especially the headache. Frequently, large volumes of CSF (>30mL) were removed without development of any complication. IDSA guidelines recommend reducing the pressure by 50% if opening pressure is “extremely high” or to a normal pressure of <20cm H2O [8]. We only continued with CSF removal if pressure remained >20cm H2O and the patient remained symptomatic, whilst checking the pressure after every 10ml CSF removal. Others recommend removing a maximum of 30 mL of CSF in order to avoid brain herniation [20] however, based on our experience, greater volumes can be drained in some patients who have had 30mL drained and still have a persistent headache and an elevated ICP >20 cm H2O in order to normalize pressure and relieve symptoms. In sub-Saharan Africa, therapeutic LPs are the mainstay in the management of raised ICP; however, still there are challenges due to limited availability of manometers [3].

In conclusion, active management of raised ICP is very important in the management of patients with cryptococcal meningitis. In resource limited settings, where lumbar drains and ventriculoperitoneal shunts are rarely placed or my lead to nosocomial infection, serial therapeutic LPs can improve the poor outcomes among patients with cryptococcal meningitis [4]. We have demonstrated that in our patient with ongoing symptoms of elevated ICP, large volumes of CSF (>30 mL) can be drained multiple times in a single day to normalize ICP to <20 cm H2O without untoward effects. More studies are needed to evaluate other innovative ways of measuring ICP since manometers are hardly available in resource limited settings. Healthcare workers need to be educated more on the role of LPs in the management of cryptococcal meningitis.

## Funding source

The parent Ambition Trial was supported via the 10.13039/501100001713European Developing Countries Clinical Trials Partnership (EDCTP), the 10.13039/100004441Swedish International Development Cooperation Agency (SIDA) (TRIA2015-1092), and the 10.13039/100010269Wellcome Trust/Medical Research Council (UK)/UKAID Joint Global Health Trials (MR/P006922/1). No extra funding was received specifically for this work.

## Consent

Written informed consent was obtained from the patient for publication of this case report.

## Declaration of competing interest

“There are none”.
